# Validity, reliability and responsiveness to change of the Italian palliative care outcome scale: a multicenter study of advanced cancer patients

**DOI:** 10.1186/s12904-016-0095-6

**Published:** 2016-02-26

**Authors:** Massimo Costantini, Elisa Rabitti, Monica Beccaro, Flavio Fusco, Carlo Peruselli, Pietro La Ciura, Alessandro Valle, Cinzia Suriani, Maria Alejandra Berardi, Danila Valenti, Felicita Mosso, Piero Morino, Giovanni Zaninetta, Giorgio Tubere, Massimo Piazza, Michele Sofia, Silvia Di Leo, Irene J. Higginson

**Affiliations:** Palliative Care Unit, Arcispedale Santa Maria Nuova-IRCCS, Reggio Emilia, Italy; Academy of Sciences of Palliative Medicine, Bentivoglio, Bologna Italy; Palliative Care Unit, ASL3 Genovese, Genoa, Italy; Palliative Care Unit, Biella Hospital, Biella, Italy; Palliative Care Unit, ASL CN1, Cuneo, Italy; FARO Foundation, Turin, Italy; Palliative Care, Distretto Vallagarina, Trento, Italy; Psycho-Oncology Unit, Istituto Scientifico Romagnolo per lo Studio e la Cura dei Tumori (IRST) IRCCS, Meldola, Italy; Palliative Care Network, AUSL, Bologna, Italy; Palliative Care Unit, ASL TO 4, Turin, Italy; Convento delle Oblate Hospice, Azienda Sanitaria, Florence, Italy; Hospice Domus Salutis, Fondazione Teresa Camplani, Brescia, Italy; Hospice ASL 1 Imperiese, Sanremo, Italy; S. Felice a Ema Hospice, Azienda Sanitaria, Florence, Italy; Palliative Care Unit, Garbagnate, Italy; Department of Palliative Care, Policy and Rehabilitation, Cicely Saunders Institute, King’s College London, London, UK; Psycho-Oncology Unit, Arcispedale Santa Maria Nuova-IRCCS, Reggio Emilia, Italy

**Keywords:** Palliative care, Outcomes, Outcome assessment, Outcome measurement, Palliative care outcome scale, POS

## Abstract

**Background:**

There is an increasing requirement to assess outcomes, but few measures have been tested for advanced medical illness. We aimed to test the validity, reliability and responsiveness of the Palliative care Outcome Scale (POS), and to analyse predictors of change after the transition to palliative care.

**Methods:**

Phase 1: multicentre, mixed method study comprising cognitive and qualitative interviews with patients and staff, cultural refinement and adaption. Phase 2: consecutive cancer patients on admission to 8 inpatient hospices and 7 home-based teams were asked to complete the POS, the EORTC QLQ-C15-PAL and the FACIT-Sp (T0), to assess internal consistency, convergent and divergent validity. After 6 days (T1) patients and staff completed the POS to assess responsiveness to change (T1-T0), and agreement between self-assessed POS and POS completed by the staff. Finally, we asked hospices an assessment 24–48 h after T1 to assess its reliability (test re-test analysis).

**Results:**

Phase I: 209 completed POS questionnaires and 29 cognitive interviews were assessed, revisions made and one item substituted. Phase II: 295 consecutive patients admitted to 15 PCTs were approached, 175 (59.3 %) were eligible, and 150 (85.7 %) consented. Consent was limited by the severity of illness in 40 % patients. We found good convergent validity, with strong and moderate correlations (r ranged 0.5–0.8) between similar items from the POS, the QLQ-C15-PAL and the FACIT-Sp. As hypothesised, the physical function subscale of QLQ-C15-PAL was not correlated with any POS item (r ranged -0.16–0.02). We found acceptable to good test re-test reliability in both versions for 6 items. We found significant clinical improvements during the first week of palliative care in 7/10 items assessed-pain, other symptoms, patient and family anxiety, information, feeling at peace and wasted time.

**Conclusions:**

Both the patient self-assessed and professional POS versions are valid and with an acceptable internal consistency. POS detected significant clinical improvements during palliative care, at a time when patients are usually expected to deteriorate. These results suggest that there is room for substantial improvement in the management of patients with advanced disease, across all key domains-symptoms, psychological, information, social and spiritual.

**Electronic supplementary material:**

The online version of this article (doi:10.1186/s12904-016-0095-6) contains supplementary material, which is available to authorized users.

## Background

Capturing the patient centred outcomes remains pivotal for demonstrating the value and quality of medical care. Outcome information is vital for evaluation, quality improvement, and to sustain services [1]. It allows patients to assess the quality of their care [2], and when captured in real time can help to screen for problems and monitor response to ensure professionals can appropriately support patients and family members [3]. In research, outcome measures are at the crux of assessing response to treatment [4]. Although the numbers of people with chronic, progressive and advanced illness are increasing [5], outcome measures in this context are lacking, yet are essential to develop appropriate interventions and support the development of models of care.

Outcome measurement in advanced illness brings particular problems. Physical function-a central component in most quality of life and outcome measures-is often not the patient’s main priority, nor is it necessarily the target of medical care. In advanced or progressive illness, many standard quality of life measures have severe floor effects, i.e., their values are always low, even if symptoms improve. This has led to an assumption that in advanced disease and towards the end of life, a patient’s quality of life cannot improve. However, an appropriate palliative care approach may lead to improvements in symptoms, psychological, social and spiritual wellbeing. Appropriate tools are needed to detect changes in these complex aspects, important for quality of life in advanced illness. At the same time, patients are too ill to complete long questionnaires and need short outcome tools. Capturing complex aspects with a short instrument is difficult. This has often led to the development of tools for proxy completion, but these have questionable validity [6].

It is crucial to build on existing tools, and yet ensure these are well validated. Harding R et al, in a European wide survey with 311 respondents in palliative care settings, identified 99 different tools being used in clinical care and audit, and 94 in research, that were cited by less than 10 participants. This makes comparison and standardisation difficult [7]. One barrier to the more widespread use of common tools is an appropriate and validated translation in different cultures and languages of existing tools [8]. Moreover, a better knowledge of the distribution of outcomes in different settings and stages of cancer disease, including the analysis of factors associated to their changes, makes possible a process of measurement and evaluation, for quality or research purpose, of different aspects of palliative care provision.

Therefore, this multicentre study aimed to culturally adapt and test the feasibility, validity, reliability and responsiveness of a brief and widely used outcome measure, the Palliative care Outcome Scale (POS), using the Italian context as a model. Moreover, we examined predictors of change on POS scales after transition to palliative care settings.

## Methods

### Study design, settings and ethical approval

This is a mixed method multicentre study, following relevant European Organisation for Research and Treatment of Cancer (EORTC) and other guidelines [9, 10]. Phase I assessed content and face validity, including preliminary testing, forward-backward translation and cognitive testing of the POS. Phase II formally evaluated the POS, testing validity, reliability, responsiveness to change, including effect size for use in clinical practice and research, and determined factors predicting response to change.

Twenty Palliative Care Teams (PCTs) from six Italian regions took part, 10 inpatient hospices (Hospice of Meldola, Forlì; Hospice of Lanzo Torinese, Torino; Hospice of Garbagnate, Milano; Hospice Seragnoli, Bologna; Hospice ASL 1 imperiese, Imperia; Hospice Fondazione FARO, Torino; Hospice Busca, Cuneo; Hospice Domus Salutis, Brescia; Hospice of Biella; Hospice G. Ghirotti, Genova), and 10 home-based PCTs (Fondazione FARO Home Care PCT, Torino; Home Care PCT, Trento; Home Care PCT Firenze Sud; Home Care PCT ASL 3 Genovese, Genova; Home Care PCT, Cuneo; Home Care PCT Firenze Centro; Home Care PCT Biella; Home Care PCT Crema; Home Care PCT Lugo, Ravenna; Home Care PCT of Firenze).

Four teams (two inpatient hospices, two home-based PCT’s) participated in the preliminary testing, 16 in cognitive interviewing and 15 in phase II (8 inpatient hospices and 7 home-based PCTs). All were multiprofessional PCTs comprising doctors, nurses, and for some teams, psychologists and/or social workers. The inpatient hospice PCTs provided in-patient care and took over provision of all aspects of care for patients and their families. The home-based PCTs supported patients and their families in the community, offering an extra layer of support for those with the most complex or advanced illness. Liaison with the PCTs was conducted primarily virtually, using e-mail, telephone and skype.

The Ethical Committee of the National Institute for Cancer Research of Genoa approved the study (Deliberation EC07.001 of 19 February 2007).

### Instruments

1. The Palliative care Outcome Scale (POS) is a widely used, validated, brief outcome measure, used in in-patient, community and outpatient settings among patients with advanced illnesses [11–13]. It was initially developed for assessing outcomes in advanced cancer patients [11] based on systematic reviews, collaboration of a multidisciplinary advisory group and input from patients and caregivers. Subsequently, the POS was used widely in many settings, contexts and among patients with different and multiple conditions. It has established validity (tested against established longer measures and qualitative reports), reliability, and internal consistency (Cronbach’s alpha ~0.7). It demonstrated responsiveness to change and acceptability, taking around 5 min to complete [2]. The original English version of the POS comprises 10 items, including physical and psychological symptoms, spiritual and emotional dimensions, communication with patients and families and practical concerns related to stage of disease. The POS also includes an open optional question to list the main concerns. Each of the 10 items is scored with a Likert scale ranging from 0 to 4, where 0 represents the best possible care and 4 the worst. POS items can be considered separately or summed in a total score ranging from 0 to 40. The 10-items original POS version 1 was valuable for use with patients with palliative care needs. POS version 2 maintains the original structure of the questionnaire, with a change in the dimension explored in item 7. In version 1 item 7 asks the patient about “life worthwhile”, in version 2 asks if the patient is feeling depressed. Both POS versions have a format for patient self-assessment, and one for proxy, professional, assessment. A more detailed description of the characteristics of the two questionnaires are available in the Palliative care Outcome Scale web page [14].
2. The 15-item European Organisation for Research and Treatment of Cancer quality of life core 15 palliative questionnaire (EORTC QLQ-C15-PAL) was developed via structured shortening of the longer core 30-item questionnaire developed to measure quality of life in cancer research [15]. The QLQ-C15-PAL includes two functional scales (physical and emotional function), seven symptom scales (fatigue, pain, nausea and vomiting, dyspnea, insomnia, appetite loss and constipation), and a single-item global health/quality-of-life scale. Higher scores in the functional scales and the global quality-of-life scale indicate better quality of life. Higher scores for the symptom scales indicate lower quality of life. The QLQ-C15-PAL takes around 20 min to complete, has good internal consistency (Cronbach’s α ≥0.7), was validated against the longer EORTC QLQ-C30 and has been shown to correlate with the Brief Pain Inventory and the Beck Depression Inventory. It can detect differences in performance status and predict survival [16, 17]. The Italian version and the Scoring Manual were provided by the EORTC, although a validation in Italian was not published. It is designed for patient self-completion.
3. The Functional Assessment of Chronic Illness Therapy, Spiritual Wellbeing Scale (FACIT-Sp) was developed originally in cancer patients to capture spiritual well-being. The instrument comprises 12 positive statements, with two subscales, one measuring a sense of meaning and peace (8 statements) and the other assessing the role of faith in illness (4 statements). Each statement is rated from 0 (not at all) to 4 (very much). A total score ranging from 0 to 48 for assessing spiritual well-being can also be produced. For both the scales and the overall score, the higher is the score; the better is the spiritual well-being. It has good internal consistency reliability, correlates with other quality of life measures and measures of religion and spirituality and takes around 5–10 min to complete [18–20]. The Italian version validation was not published, but the FACITOrg provided the Italian version of the questionnaire with the Scoring Manual. It is designed for patient self-completion.

### Procedures and participants

#### Phase I

- **Preliminary field-testing and assessment of feasibility, content and face validity**, using version 1 for staff, previously translated, but not back translated, by Franco Toscani [21]. We asked palliative care clinicians in four PCTs to assess all new patients using this version of the POS during their weekly meetings. For each item, staff assessed the comprehensibility, face validity relative to the assessed dimension, uniqueness and the relevance of the content and rating scale. They reported any other dimensions not covered by the POS potentially useful for assessing quality of life in that specific patient.
- **Translation** into Italian the original English POS (version 2, both staff and patient completed questionnaires) following the forward-backward procedures recommended by the EORTC QL Group [9]. This was combined with findings from the preliminary testing to provide a version of POS for cognitive testing.
- **Cognitive testing** of the POS in 16 PCTs. Clinicians were requested to identify two patients in their care and asked them to complete the POS. After completion, the clinicians conducted a semi-structured interview with patients, focused on any potential problems in filling in each item of the POS. Following the EORTC [9] guidelines, patients were asked to respond to five questions for each item of the POS:
  - Did you have difficulty in replying to this question?
  - Did you find this question unclear?
  - Were words in this question that you found difficult to understand?
  - Did you find the way was worded to be upsetting or annoying in any way?
  - Would you have asked the question in a different way?

The comments were transcribed verbatim during the interview. The interviewer grouped the transcripts according to the five questions, and sent the material to the coordination centre. A researcher (MC) reviewed the written material and identified all relevant issues for each specific question. The POS was then modified accordingly for formal testing.

#### Phase II

We included consecutive consenting cancer patients admitted to the care of the participating PCTs, and excluded patients unable or unwilling to provide informed consent. Reasons for exclusion and refusals were recorded. Patients completed the POS at admission (T0) together with the EORTC QLQ-C15-PALand the FACIT-Sp. At admission, the staff also collected demographic and clinical details, including the functional status through the Eastern Cooperative Oncology Group (ECOG) scale. After 6 days from admission (T1), patients were requested to complete again the POS. In the meantime, the patient’s main clinician, blind to the patient scoring, was requested to complete the staff version of the POS.

To assess test-retest reliability of the POS, we asked inpatient hospices an additional POS assessment 24–48 h after T1 assessment (T2). Also in this step, the clinician completed a staff POS assessment blind to the patient scoring. We limited this assessment to the inpatient hospices because of the greater intensity of contact and care of their professionals. The short time to retest (24–48 h) was a compromise between the need to avoid recall bias and the need to retest patients in a stable clinical condition [22].

### Statistical analysis

We evaluated the psychometric properties of the POS according to standard methods [9, 23].

1. Feasibility and acceptability, data and scaling quality, was assessed by calculating the percentage of missing items (number of missing items/total number of item responses possible), the distribution of scores and floor or ceiling effects. We assumed that 5 % was an acceptable proportion of missing for each item of the questionnaire, taking into account the settings where the POS was administered.
2. Construct validity was assessed by determining correlations between POS items and similar items on QLQ-C15-PAL and FACIT-Sp. We developed predetermined hypotheses of strong and moderate convergent validity (for similar items) and divergent validity (for diverse items). We hypothesized:
  very similar items would have strong correlations (>0.7)-POS pain vs. QLQ-C15-PAL pain;
  items with some similar underlying constructs would have moderate correlations (0.4–0.7)-POS anxiety and depression vs. QLQ-C15-PAL emotional functioning; POS other symptoms vs. QLQ-C15-PAL individual symptoms; POS ‘at peace’ vs. FACIT-Sp meaning and peace subscale, and FACIT total;
  items with different constructs would have very low correlations (<0.2), for example all POS items should have a low correlation with all QLQ-C15-PAL physical functioning scale;
3. Test-retest reliability was assessed by testing for percentage agreement, percentage agreement within one score, non-parametric correlation, and weighted Cohen’s Kappa, which controls for chance agreement, between POS scores assessed twice over a short period (the first at T1 and the second at T2, 24–48 h later), assuming that the quality of life of these patients should be rather stable. Kappa should be interpreted with caution when many patients score similar values. Agreement for the POS total score was assessed by estimating one-way random Intraclass Correlation Coefficient (ICC) [24].
4. Internal consistency of the scales was determined using Cronbach’s alpha coefficient. Internal consistency is valid only if all items form a unidimensional (sub) scale, and the set of items forms a reflective model (i.e., all items are expected to change when the construct changes). According to the criteria proposed by Terwee CB, et al [23], Cronbach’s alpha ≥0.70 indicates good internal consistency without homogeneity.
5. Responsiveness to change was determined by determining the change in POS score from admission (T0) to the subsequent assessment (6 ± 2 day later-T1). We calculated mean scores and effect sizes.
6. Clinician (doctor or nurse) proxy assessments were tested for validity by comparing their scores with patient (as the gold standard) synchronous (±1 day) scores. The assessment was assessed at T1. We calculated the percentage agreement, percentage agreement within one score, weighted Kohen’s Kappa, correlations. We also estimated the one-way ICC for the POS total scores [24].

A linear regression analysis was flitted to the data, using the change from admission as continuous dependent variable. The aim of this analysis was to identify subgroups of patients with specific characteristics showing significant improvement or deterioration in their quality of life in the week after admission to palliative care. We tested the association between the dependent variable and the demographic and clinical characteristics of the patients (age, gender, education, marital status, primary tumour, ECOG, setting) in both univariate and multivariate analyses. In the multivariate analysis, we estimated the means of the changes from admission, from the regression model after adjusting for all independent variables.

All analyses were performed using SPSS v. 20 (IBM SPSS Inc., Chicago, IL).

## Results

### Phase I, feasibility, content and face validity, cultural adaptation

For the preliminary assessment, the POS was completed during staff meetings for 82 patients, giving 209 completed questionnaires (most patients had 2–3 assessments). For 96 (46 %) evaluations, the staff did not identify any problems/issues. For 113 (54 %) concerns in comprehensibility and/or uniqueness of the content were reported [see Additional file 1]. Most (*n* = 57, 50 %) were related to item 5 (information), in particular variation of information levels between patients and families. In all assessments, staff deemed the POS dimensions as comprehensive, except for one case, which proposed an additional assessment of communication with other professionals. Clinicians reported that a POS manual providing further guidance would be helpful.

Results of translation, back-translation and adaptation of the Italian version of the POS patient version are summarized in Additional file 2. This includes modifications made as a result of the preliminary assessment’s findings. The main change was that we replaced the original Item 8 (“Have you felt good about yourself as a person?”) with the question: “Are you at peace?”, originally developed and validated to probe spiritual concerns at the end of life [25] and used in the African adaption of POS [26].

In the cognitive testing 29 questionnaires (range 1–4 per PCT) were administered, including 15 patients. Overall, the items performed well [see Additional file 3] without any major difficulties for seven out of the ten items. Some patients found it difficult to answer items 6 (share feelings) and 10 (personal affairs). One patient reported “… difficulties due to the problematic relationship with my family” for item 6. Only one item (number 10) was found upsetting by only one patient, who “… found the question too intrusive”. Patients experienced most difficulties in answering item 8 (are you feel at peace?), but we were not able to find a better alternative. Based on these results no further changes were made.

### Phase II formal evaluation

**Fig. 1:**
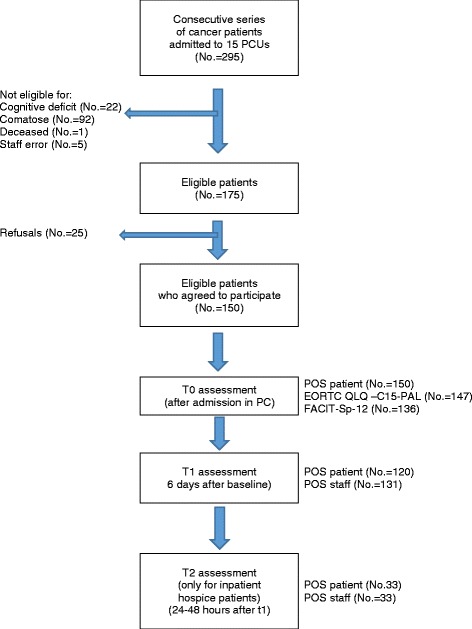
Flow chart of the study

Two hundred and ninety-five consecutive cancer patients admitted to 15 PCTs were screened for their eligibility, of these 175 (59.3 %) resulted eligible. Main reasons for exclusion were coma and cognitive impairment (Fig. 1). Participation to the study was proposed to 175 patients, and 150 (85.7 %) consented. The characteristics of the 150 eligible consenting patients were similar to the whole sample in terms of age, gender, educational level, diagnosis and marital status, but had less severe functional status as determined by ECOG status (*P* < 0.001) (Table 1). Fewer patients from inpatient hospices than home based PCTs were able to be included in the study. (*P* = 0.002) All 150 patients completed the POS at admission, slightly lower numbers for QLQ-C15-PAL and FACIT-Sp (Fig. 1). After 6 days at T1, 138 patients were alive and 120 (87 %) POS patient and 131 (95 %) POS staff assessments were completed. At T2, in the 8 inpatient hospices that reassessed the patients, 59 patients were alive and 33 (56 %) POS patient and staff (Fig. 1) assessments were completed.

**Table 1:** Characteristics of the whole sample and of eligible patients

	Whole sample	Eligible		*P*-value
	N	N	%	
Age at death (years)				
18–55	32	18	56.2	
56–65	53	28	52.8	
66–75	84	45	53.6	
76–85	87	46	52.9	
> 85	39	13	33.3	0.228
Gender				
Males	160	77	48.1	
Females	131	73	55.7	0.197
Unknown	4	*-*		
Education (years)				
≤ 5	146	74	50.7	
6–8	76	37	48.7	
9+	58	35	60.3	0.358
Unknown	15	4		
Marital status				
Single	85	45	52.9	
Married	182	91	50.0	0.654
Unknown	28	*14*		
Primary tumor				
Head and neck	8	4	50.0	
Digestive system	114	64	56.1	
Respiratory system	73	35	47.9	
Breast	24	14	58.3	
Genitourinary system	34	19	55.9	
Hematological	12	6	50.0	
Others	30	8	26.7	0.151
ECOG				
Fully active	5	4	80.0	
Restricted	10	9	90.0	
Ambulatory	23	17	73.9	
Limited self-care	124	88	71.0	
Completely disabled	132	32	24.2	<0.001
Unknown	1	*-*		
Settings				
In-patient hospice PCTs	160	68	42.5	
Home based PCTs	135	82	60.7	0.002
Totals	295	150	50.8	

*ECOG* eastern cooperative oncology group, *PCTs* palliative care teams

There was little missing data for POS assessments, all less than 5 %. The highest was for the items ‘information’ and ‘personal affairs’ (3.3 % each) [see Additional file 4]. The entire range of possible scoring was used for all POS items. ‘Family anxiety’ had the highest proportion of score 4 (worst score), with 49.7 % of recording that their families were overwhelmingly anxious. For four items-‘wasted time’, ‘personal affairs’, ‘information’, ‘share feelings’-were most commonly no problem (score 0), recorded by 59–80 % of patients. The last four items, plus ‘other symptoms’ and ‘family anxiety’ were poorly correlated with the remaining items of the POS scale, with corrected item-total correlations below 0.40 (between 0.17 and 0.35). Cronbach’s alpha (95 % CI) for the 10 POS items at admission was 0.67 (0.59–0.73).

Correlations between POS and QLQ-C15-PAL and FACIT-Sp met most the prior hypotheses (Table 2). There was a strong correlation between the items assessing pain (*r* = 0.77), and moderate correlations between POS anxiety and depression and QLQ-C15-PAL emotional functioning (*r* = -0.51 and−0.68, respectively). We also found moderate correlations between POS ‘at peace’ and the FACIT-Sp meaning and peace subscale (*r* =−0.44) and the total score (*r* =−0.40), but not the ‘faith’ subscale (*r* =−0.16). POS ‘other symptoms’ was correlated moderately with fatigue, nausea and vomiting, appetite loss and constipation on QLQ-C15-PAL, but not breathlessness. As hypothesised, the physical function subscale of QLQ-C15-PAL was not correlated with any POS item (r ranged−0.16 to 0.02), nor with the total POS score (*r* =−0.12).

**Table 2:** Correlation between POS and EORTC QLQ-C15 PAL and the Spiritual scale of the FACIT at admission

	**POS**	Pain	Other Symptoms	Anxiety	Family anxiety	Information	Share feelings	Depression	Feeling at peace	Wasted time	Personal affairs
**EORTC QLQ -C15-PAL**											
**Functional scales**											
Physical Functioning	−.12	−.09	−.16	−.03	−.13	−.04	.01	−.16	−.05	−.03	.02
Emotional Functioning	**−.57**	**−.35**	**−.23**	**−.51**	−.12	−.25	.01	**−.68**	**−.41**	**−.24**	−.05
**Symptom scales**											
Fatigue	**.30**	.18	**.30**	.15	.17	.16	.09	**.22**	−.01	.05	−.01
Nausea and vomiting	**.33**	.11	**.41**	.14	.17	.12	.04	.19	.06	.16	.01
Pain	**.54**	**.77**	.08	**.24**	**.22**	.20	.14	**.25**	.19	**.26**	.11
Dyspnoea	.13	−.02	.04	.12	.05	.17	.02	.01	.07	.11	.13
Insomnia	**.30**	.16	.18	**.28**	.15	.01	−.04	.23	.13	−.04	−.01
Appetite loss	.06	−.03	**.32**	.08	**.22**	−.02	−.01	.13	−.12	−.02	**−.29**
Constipation	.16	.07	**.26**	.02	.15	.12	.01	.11	.01	.10	.01
Global health status/QOL	**−.23**	**−.27**	−.04	−.12	−.18	−.15	−.05	−.13	−.08	−.10	−.06
**FACIT-Sp-12**											
FACIT 1	**−.46**	**−.27**	.05	**−.43**	.02	−.20	**−.33**	**−.41**	**−.44**	−.13	−.15
FACIT 2	−.24	−.07	.00	−.08	−.02	−.04	−.18	−.15	−.16	−.03	−.20
FACIT TOTAL	**−.42**	**−.26**	.10	**−.32**	.05	−.18	**−.33**	**−.31**	**−.40**	−.11	−.23

Statistical significant correlations (*P*-values <0.01) were highlighted

*EORTC QLQ -C15-PAL* European Organisation for Research and Treatment of Cancer Quality of Life core 15 palliative Questionnaire

*FACIT-Sp-12* Functional Assessment of Chronic Illness Therapy, spiritual wellbeing scale

*POS* palliative care outcome scale

Test re-test reliability of the POS total score for both versions (self-assessed by the patients and assessed by the staff) was rather good, with the ICC of 0,72 (95 % CI = 0,50–0,85) and 0,82 (95 % CI = 0,67–0,91) respectively. Test re-test reliability of the POS items for both versions showed acceptable or good agreement over time for all items apart item 2 (other symptoms) and 6 (share feelings) when assessed by the patients, and items 9 (wasted time) and 10 (personal affairs). These results are difficult to judge accurately because it was difficult to identify and ensure patients were stable. (Tables 3 and 4).

**Table 3:** Test re-test: agreement of the POS scores self-assessed by the patients at T1 and 24–48 h later (T2)^a^

	Patients T1	Patients T2	Agreement				
	Mean (SD)	Mean (SD)	Agreement (%)	Agreement within one score (%)	Weighted kappa (95 % CI)		Spearman correlation
Pain	1.5 (1.1)	1.4 (0.9)	51.5	90.9	0.46	(0.27–0.65)	0.66
Other symptoms	1.4 (1.1)	1.5 (1.1)	24.2	75.8	0.12	(0.00–0.35)	0.20
Anxiety	1.6 (1.2)	1.5 (1.1)	48.5	87.9	0.50	(0.30–0.69)	0.68
Family anxiety	2.9 (1.3)	2.4 (1.3)	48.5	81.8	0.44	(0.20–0.68)	0.59
Information	0.6 (1.1)	0.6 (1.1)	78.1	84.8	0.53	(0.29–0.77)	0.79
Share feelings	1.0 (1.1)	0.9 (1.0)	36.4	72.7	0.04	(0.00–0.29)	0.03
Depression	1.9 (1.5)	1.4 (1.3)	48.5	75.8	0.47	(0.26–0.68)	0.59
Feeling at peace	1.2 (1.0)	1.1 (1.1)	57.6	90.9	0.55	(0.36–0.74)	0.68
Wasted time	0.3 (1.0)	0.2 (0.8)	84.8	84.8	0.16	(0.00–0.50)	0.27
Personal affairs	0.4 (1.0)	0.5 (1.2)	72.7	72.7	0.12	(0.00–0.42)	0.17
POS total score	12.7 (6.2)	11.7 (6.0)	-	-	0.72^b^	(0.50–0.85)	0.72

*POS* palliative care outcome scale

^a^based on a sample of 33 inpatient hospices patients

^b^one-way Intraclass Correlation Coefficient (ICC)

**Table 4:** Test re-test: agreement of the POS scores assessed by the staff at T1 and 24–48 h later (T2)^a^

	Patients T1	Patients T2	Agreement				
	Mean (SD)	Mean (SD)	Agreement (%)	Agreement within one score (%)	Weighted kappa (95 % CI)		Spearman correlation
Pain	1.0 (0.9)	1.1 (1.0)	57.6	90.9	0.50	(0.29–0.71)	0.61
Other symptoms	1.5 (1.2)	1.4 (1.1)	42.4	93.9	0.49	(0.30–0.68)	0.67
Anxiety	1.8 (1.3)	1.6 (1.3)	63.6	87.9	0.67	(0.49–0.84)	0.80
Family anxiety	2.3 (1.6)	2.1 (1.5)	48.5	78.8	0.54	(0.34–0.75)	0.66
Information	0.9 (1.3)	0.8 (1.4)	75.8	90.9	0.73	(0.54–0.93)	0.70
Share feelings	1.1 (1.2)	0.9 (1.1)	57.6	72.7	0.38	(0.10–0.66)	0.36
Depression	1.8 (1.3)	2.0 (1.2)	57.6	90.9	0.63	(0.46–0.81)	0.79
Feeling at peace	1.5 (1.0)	1.4 (1.0)	39.4	97.0	0.34	(0.15–0.53)	0.54
Wasted time	0.1 (0.5)	0.1 (0.5)	87.9	87.9	NE	NE	−0.06
Personal affairs	0.6 (1.2)	0.2 (0.8)	75.8	75.8	0.24	(0.01–0.48)	0.48
POS total score	12.6 (6.0)	11.8 (6.0)	-	-	0.82^b^	(0.67–0.91)	0.83

*NE* not estimable because observed agreement is smaller than expected

*POS* palliative care outcome scale

^a^based on a sample of 33 inpatient hospices patients

^b^one-way Intraclass Correlation Coefficient (ICC)

**Fig. 2:**
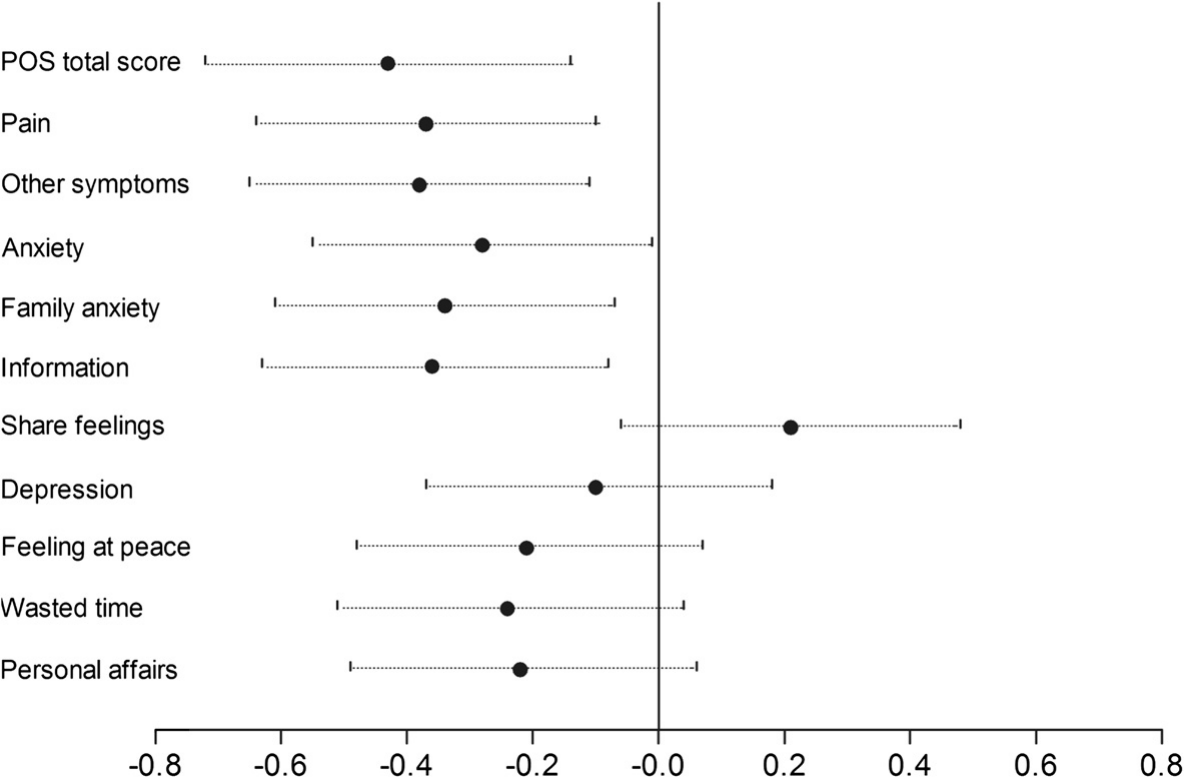
Effect sizes (95 % CI) of the change in the POS scores from admission in palliative care (T0) to 6 days later (T1)

Considering responsiveness to change, we found that POS demonstrated good responsiveness to change during admission to palliative care. After 6 days from admission to PCTs, patients reported significant improvements in 7/10 POS items, all except depression, share feelings, and personal affairs (Table 5). Effect sizes for these seven items ranged between−0.21 (feeling at peace) to−0.38 (other symptoms), with a total score effect size of−0.43. (Table 5; Fig. 2).

**Table 5:** Responsiveness to change in a sample of patients self-assessed with the POS after 6 days from admission in palliative care (T0)

	No.	Admission (T0)	6 ± 2 days after T0	Difference (T1-T0)		Effect size (95 % CI)	
		Mean (SD)	Mean (SD)	Mean (95 %)			
Pain	107	1.7 (1.4)	1.2 (1.1)	−0.46	(-0.7 and -0.2)	−0.37	(−0.64 and−0.10)
Other symptoms	107	1.8 (1.1)	1.3 (1.1)	−0.42	(-0.7 and -0.2)	−0.38	(−0.65 and−0.11)
Anxiety	106	2.1 (1.3)	1.7 (1.2)	−0.35	(-0.6 and -0.1)	−0.28	(−0.55 and−0.01)
Family anxiety	107	3.0 (1.2)	2.6 (1.2)	−0.41	(-0.7 and -0.1)	−0.34	(−0.61 and−0.07)
Information	103	0.8 (1.2)	0.5 (0.9)	−0.38	(-0.6 and -0.1)	−0.36	(−0.63 and−0.08)
Share feelings	106	0.6 (0.9)	0.8 (1.0)	0.20	(-0.1 and 0.4)	0.21	(−0.06 and 0.48)
Depression	104	1.9 (1.3)	1.7 (1.2)	−0.11	(-0.3 and 0.1)	−0.10	(−0.37 and 0.18)
Feeling at peace	104	1.4 (1.2)	1.1 (1.1)	−0.24	(-0.4 and -0.1)	−0.21	(−0.48 and 0.07)
Wasted time	105	0.6 (1.3)	0.3 (1.0)	−0.27	(-0.5 and -0.1)	−0.24	(−0.51 and 0.04)
Personal affairs	103	0.5 (1.1)	0.3 (0.8)	−0.21	(-0.5 and 0.3)	−0.22	(−0.49 and 0.06)
POS Total Score	94	14.3 (6.1)	11.7 (5.7)	−2.55	(-3.7 and -1.4)	−0.43	(−0.72 and−0.14)

*POS* palliative care outcome scale

Regression analyses found two variables significantly associated with change from admission. An inverse linear relationship between years of education and improvement in POS scores after admission was observed both in univariate (*P*-value =0.008) and in multivariate analysis (*P*-value = 0.002). Then, in the multivariate analysis, the improvement was significantly (*P*-value =0.014) higher for patients admitted to Home Care as compared to those admitted to the in-patient hospice (Table 6).

**Table 6:** Association between demographic and clinical characteristics of the patients and changes in POS total scores after six days from admission in palliative care

	No.	Univariate linear regression		Adjusted linear regression^a^	
		Mean change^b^		Mean change^b^	
Age at death (years)					
18–65	31	−2.4		−3.1	
66–75	33	−2.3		−1.0	
> 75	43	−2.7	*P* = 0.779	−1.1	*P* = 0.199
Gender					
Males	55	−3.2		−2.9	
Females	52	−1.8	*P* = 0.168	−0.6	*P* = 0.069
Education (years)					
≤ 5	52	−3.8		−3.9	
6-8	28	−1.6		−1.6	
9+	26	−0.6	*P* = 0.008	−0.3	*P* = 0.002
Marital status					
Single	31	−2.0		−1.8	
Married	70	−2.4	*P* = 0.693	−1.6	*P* = 0.680
Primary tumor					
Digestive system	42	−2.0		−0.9	
Respiratory system	27	−2.3		−0.8	
Breast	10	−0.9		−1.7	
Genitourinary system	16	−4.6		−4.2	
Others	12	−3.2	*P* = 0.389	−1.2	*P* = 0.202
ECOG					
Fully active - ambulatory	23	−3.0		−2.9	
Limited self-care	67	−2.7		−2.0	
Completely disabled	17	−1.0	*P* = 0.288	−0.4	*P* = 0.109
Settings					
In-patient hospice PCTs	46	−1.3		−0.5	
Home based PCTs	61	−3.4	*P* = 0.050	−3.0	*P* = 0.014
Totals	107				

*PCTs* palliative care teams

*ECOG* eastern cooperative oncology group

*POS* palliative care outcome scale

^a^adjusted for all variables of the table

^b^a negative mean change means an improvement in quality of life six days after admission in palliative care (T1-T0) measured with the POS total scores

Comparing the staff assessments of POS vs. patients self-assessments we found moderate agreement for the POS total scores (ICC = 0,56; 95 % CI = 0,41–0,68). A good agreement (kappa > 0.40) was observed for items 1 (pain) and 2 (other symptoms), and low-moderate agreement (kappa between 0.20 and 0.40) for all the other items. Wasted time and personal affairs had strong agreement (>75 % same score), but lower Kappa because of the high percentage of 0 scores [see Additional file 5].

The staff version of POS at T1 had similar internal consistency to that found for the patient version; the staff Cronbach’s alpha (95 % CI) at T1 was 0.68 (0.61-0.75) and the patient Cronbach’s alpha (95 % CI) at T1 was 0.72 (0.65–0.78).

## Discussion

We found that the POS was a feasible opportunity to assess outcomes and quality of life in advanced illness. The professional completion of POS was possible for most patients in care, but in this sample, on admission, the severity of illness limited the self-assessment in almost 40 % of patients (115/295), due to cognitive impairment, coma or early death. We found the POS had an excellent construct validity, a limited, but acceptable internal consistency and reliability, a good convergent and divergent validity when compared with other measures. Although the professional assessments had acceptable agreement and correlation with patient ratings, there were differences, suggesting that wherever possible patient assessments should be used. The POS was responsive to change, with significant clinical improvements during the first week of palliative care in seven out of 10 items assessed - pain, other symptoms, patient and family anxiety, information, feeling at peace and wasted time.

This study detected substantial clinical improvement, even with one week of palliative care, in many dimensions important in quality of life at a point of the trajectory of disease when modern medicine often considers there is nothing more that can be done. There was a medium effect size of 0.43 for the total score and between 0.21 and 0.38 (small to medium) effect sizes for the 7/10 individual items where significant improvements were found [27]. A question is-could these benefits be achieved at an earlier time? The mean POS score at admission was 14.3 indicating that most patients had serious and multiple problems.

The regression analysis found that the improvements were six times greater in the home care group, compared to the inpatient hospice group. These findings are difficult to interpret and different explanations could be discussed. Survival is usually shorter for hospice patients, and it is possible that an earlier admission to palliative care for home care patients was associated with a greater improvement in POS total scores. Although data from recent trials of early palliative care [28–30], make this hypothesis appealing, this study does not allow to get to a clear conclusion.

An unexpected finding was the strong inverse relationship between educational level and improvement in POS score during palliative care. Recent commentaries [31] and specific researches [32] have highlighted inequities in access to palliative care across the UK, as it is less likely to be available for people living in areas of social deprivation. The results of this study, if confirmed, suggest that palliative care has the potential to benefit more just the socially disadvantaged groups.

The first four items of POS (pain, other symptoms, patients’ and family anxiety) worked well, without concerns from patients. These domains are reported by clinicians to be the most important questions [13]. However, one patient suggested replacing item 2 with a checklist of symptoms. Symptom management is a cornerstone in palliative care. Correlations with QLQ-C15-PAL found that the single POS item ‘other symptoms’ captured some symptoms, but not others, especially not breathlessness. As symptoms are highly prevalent in advanced disease, assessing individual symptoms should be considered in future developments of the POS.

We decided to change the original POS item 8, “Have you felt good about yourself”, with the Steinhauser’s item “are you at peace” that showed promising psychometric properties in the validation study [25]. The original item had experienced some problem in the validation of the German version of the POS [12]. In the African POS, the “peace item” was included in the final questionnaire [26] and subsequently showed good validity in the African context [33]. The “peace item” may cover a different dimension as compared to the removed item. This could be a limitation, as the original item 8 together with item 7 (depression) have shown to be useful for screening of depression [34].

Not all the items showed satisfactory psychometric properties. A clear floor effect was observed for items 9 “wasted time” and 10 “personal affairs”, and to a lesser extent for items 5 “information” and 6 “share feelings”. Four of these items-“other symptoms”, “share feelings”, “wasted time” and “personal affairs-" showed a poor reliability when assessed by the patients, and to a lesser extent when assessed by the staff. The low reliability of “wasted time” and “personal affairs" could be explained by the skewness of their distribution, considering that the proportion of agreement is rather high, and the kappa statistics is rather sensitive to marginal distributions. Conversely, the poor reliability of “other symptoms” and “share feelings”, associated with a wide distribution of the scores and a low proportion of agreement, suggests some problem in the items when they are self-assessed by the patients. Future revision of the tool should take into consideration these points.

This study has limitations. First, the psychometrics properties of the EORTC QLQ-C15Pal and the Facit-Sp were never assessed in Italy. Both questionnaires are widely used, and we used the official translations, but we must take into account that the results of the convergent and divergent analyses could be slightly biased. Then, we estimated test-retest reliability without any confirmation of clinical stability before retesting the patients 24–48 h later. Hospice patients are prone to deteriorate rather quickly and the inclusion of clinically instable patients could have affected the results, by providing biased underestimates of reliability. Moreover, as we did not collect any external clinical or patient-based data during prospective assessments, we could not estimate the minimal important difference for the POS. Other studies should explore this important property of the tool.

Then, we only included patients with a diagnosis of cancer, although many were elderly and had co-morbidities. The advanced phase of illness in cancer is common in internal medicine, and there are many similarities in symptoms and problems between cancer and non-cancer [30]. Five percent of patients declined to take part in the study. Although this is low, we do not know whether they refused the study, with all the questionnaires, or completion of the POS in itself.

Finally, the study was based on one country, Italy, where the provision of services may be different to others; but we had six regions and 20 centres that should guarantee a certain degree of heterogeneity.

## Conclusions

The POS covers all key dimensions for assessing quality of life in palliative care among patients with advanced disease. No other relevant dimensions emerged in this large multicentre study, although we identified that the ‘other symptoms’ item would benefit from being divided into some individual symptoms. The two versions of POS-for patient self-completion and for staff completion-are valid and reliable. POS detected significant clinical improvements during 1 week of palliative care, at a time when patients are usually expected to deteriorate. The effect size was 0.4 for the total score. These results suggest that there is room for substantial improvement in the management of patients with advanced disease in hospitals and in the community, across all key domains-symptoms, psychological, information, social and spiritual.

## Additional files

Additional file 1:
**Problems encountered by the Palliative Care professionals in filling in the POS (version 1 for the staff).** (DOCX 21 kb) Additional file 2:
**Changes performed in the translation into Italian of the POS (version 2 for the patient).** (DOCX 21 kb) Additional file 3:
**Pilot test of the Italian POS (version 2 for the patient) in a sample of 29 patients from 15 PCTs.** (DOCX 22 kb) Additional file 4:
**Missing values, floor-ceiling effect and internal consistency of the POS scale in the sample of 150 patients assessed with POS at admission.** (DOCX 19 kb) Additional file 5:
**Agreement POS scores self-assessed by patients and assessed by staff at T1 (6 days after admission).** (DOCX 19 kb)

## Abbreviations

ECOG: Eastern Cooperative Oncology Group
EORTC: European Organisation for Research and Treatment of Cancer
EORTC QLQ-C15-PAL: European Organisation for Research and Treatment of Cancer quality of life core 15 palliative questionnaire
EORTC QLQ-C30: European Organisation for Research and Treatment of Cancer quality of life core 30 questionnaire
FACIT-Sp: functional assessment of chronic illness therapy, spiritual wellbeing scale
PCT: Palliative Care Team
POS: palliative care outcome scale
