# Supplementary material for: Validity, reliability and responsiveness to change of the Italian palliative care outcome scale: a multicenter study of advanced cancer patients
Source: BMC Palliat Care. 2016 Feb 26;15:23. doi: 10.1186/s12904-016-0095-6 (PMC4768331; doi:10.1186/s12904-016-0095-6)
Supplement: Additional file 1: — Problems encountered by the Palliative Care professionals in filling in the POS (version 1 for the staff). (DOCX 21 kb) [file 12904_2016_95_MOESM1_ESM.docx]

Additional file 1: Problems encountered by the Palliative Care professionals in filling in the POS (version 1 for the staff)

| **ITEM** | **QUESTION** | **RESPONSE SCALE** |
| --- | --- | --- |
| 1. Pain | No specific problems | No specific problems |
| 2. Other symptoms | Not clear if it includes psychological symptoms | Difficult to score in patients with multiple symptoms |
| 3. Anxiety | No specific problems | No specific problems |
| 4. Family anxiety | No specific problems | Problems to respond for different levels of anxiety in different family members (the worst case? the situation of the primary caregiver?) |
| 5. Information | Information might be different   - for the patient and the family - for diagnosis, prognosis and treatments | Need to specify what type of information it refers (diagnosis, prognosis, therapy, etc...) , and difficult to score whether the patient does not ask information and is satisfied for that |
| 6. Share feelings | How to consider patients sharing how they are feeling with professionals? It can occur with or without sharing with families or friends. | No specific problems |
| 7. Life worthwhile | The content of “life was worth living” should be better defined | No specific problems |
| 8. Self-worth | The content of “felt good about themselves” should be better defined | No specific problems |
| 9. Wasted time | Problems might occur also to the family | No specific problems |
| 10. Personal affairs | Need to distinguish types of the problems | No specific problems |
|  |  |  |

POS=Palliative care Outcome Scale
