# Supplementary material for: Validity, reliability and responsiveness to change of the Italian palliative care outcome scale: a multicenter study of advanced cancer patients
Source: BMC Palliat Care. 2016 Feb 26;15:23. doi: 10.1186/s12904-016-0095-6 (PMC4768331; doi:10.1186/s12904-016-0095-6)
Supplement: Additional file 2: — Changes performed in the translation into Italian of the POS (version 2 for the patient). (DOCX 21 kb) [file 12904_2016_95_MOESM2_ESM.docx]

Additional file 2: Changes performed in the translation into Italian of the POS (version 2 for the patient)

| **ITEM** | **Original English POS** | **Modified Italian POS** |
| --- | --- | --- |
| 1. Pain | … have you been affected by pain? | … have pain been affecting how you feel? |
| 2. Other symptoms | … have other symptoms (e.g. feeling sick, having a cough or constipation) been affecting how you feel? | No changes |
| 3. Anxiety | …. have you been feeling anxious or worried about your illness or treatment? | No changes |
| 4. Family anxiety | … have any of your family or friends been anxious or worried about you? | No changes |
| 5. Information | … how much information have you and your family or friends been given?  3. Very little given and some questions were avoided | … how much information have you been given?  3. Very little information given and some questions have not been answered |
| 6. Share feelings | … have you been able to share how you are feeling with your family or friends? | No changes |
| 7. Depression | … have you been feeling depressed? | No changes |
| 8. Self-worth 🡪 feeling at peace | … have you felt good about yourself as a person? | … are you at peace? |
| 9. Wasted time | … how much time do you feel you have wasted on appointments relating to your healthcare, e.g. waiting around for transport or having the same tests repeated? | No changes |
| 10. Personal affairs | … have any practical matters resulting from your illness, either financial or personal, been addressed? | No changes |

POS=Palliative care Outcome Scale
