# Supplementary material for: Validity, reliability and responsiveness to change of the Italian palliative care outcome scale: a multicenter study of advanced cancer patients
Source: BMC Palliat Care. 2016 Feb 26;15:23. doi: 10.1186/s12904-016-0095-6 (PMC4768331; doi:10.1186/s12904-016-0095-6)
Supplement: Additional file 3: — Pilot test of the Italian POS (version 2 for the patient) in a sample of 29 patients from 15 PCTs. (DOCX 22 kb) [file 12904_2016_95_MOESM3_ESM.docx]

Additional file 3: Pilot test of the Italian POS (version 2 for the patient) in a sample of 29 patients from 15 PCTs

|  | **Difficulty** | **Clarity** | **Understanding** | **Upsetting** | **suggestions**  **for a different formulation** | **Suggested changes in items’ formulation** |
| --- | --- | --- | --- | --- | --- | --- |
| 1. Pain | - | - | 1 | - | 1 | - difficulty to understand “severe”. I’d specified: “… has influenced the behaviour, reasoning ... at certain times of the day” |
| 2. Other symptoms | - | 1 | 2 | - | 2 | - difficulty to understand “severe”, I would replace it with "serious" - it’s unclear. Better listing all the symptoms |
| 3. Anxiety | 1 | 1 | - | - | 2 | - suggestion to split it in disease and therapies” |
| 4. Family anxiety | 2 | 1  (maybe) | - | - | 3 | - I’d remove “it seems to affect their concentration” - I’d not use “occasionally” |
| 5. Information | 1 | 2 | - | - | 4 | - Suggestions to explain in what context or circumstance I have been given little information" |
| 6. Share feelings | 3 | 1 | 1 | - | 3 | - emotional difficulties due to the problematic relationship with my family” |
| 7. Depression | - | - | - | - | 2 | - depression is a strong word, suggestions to use alternative words such as “ worried " |
| 8. Feeling at peace | 9 | 5  (1 maybe) | 3 | - | 6  (1 maybe) | - suggestions to use alternative wording: "There is something that you will would sort out?" or "Do you have some suspended?" |
| 9. Wasted time | 1 | 1 | 1 | - | 1 | - I’d put more answer choice" |
| 10. Personal affairs | 4 | 3 | 2 | 1 | 4 | - A patient found the item too intrusive. - Suggestions to better specify" financial " |

POS=Palliative care Outcome Scale
