# Supplementary material for: Validity, reliability and responsiveness to change of the Italian palliative care outcome scale: a multicenter study of advanced cancer patients
Source: BMC Palliat Care. 2016 Feb 26;15:23. doi: 10.1186/s12904-016-0095-6 (PMC4768331; doi:10.1186/s12904-016-0095-6)
Supplement: Additional file 4: — Missing values, floor-ceiling effect and internal consistency of the POS scale in the sample of 150 patients assessed with POS at admission. (DOCX 19 kb) [file 12904_2016_95_MOESM4_ESM.docx]

Additional file 4: Missing values, floor-ceiling effect and internal consistency of the POS scale in the sample of 150 patients assessed with POS at admission

|  | Missing | |  | Floor-ceiling effect | |  | Internal consistency | |
| --- | --- | --- | --- | --- | --- | --- | --- | --- |
|  | No. (%) | |  | score 0  % | Score 4  % |  | Corrected  item-total  correlation | Cronbach’s  Alpha if item deleted |
|  |  |  |  |  |  |  |  |  |
| Pain | 150 | – |  | 30,0 | 8,7 |  | 0,43 | 0,63 |
| Other symptoms | 150 | – |  | 18,7 | 4,7 |  | 0,25 | 0,66 |
| Anxiety | 148 | (1,3) |  | 16,9 | 14,9 |  | 0,49 | 0,62 |
| Family anxiety | 149 | (0,7) |  | 5,4 | 49,7 |  | 0,20 | 0,67 |
| Information | 145 | (3,3) |  | 60,0 | 5,5 |  | 0,35 | 0,65 |
| Share feelings | 149 | (0,7) |  | 59,1 | 2,0 |  | 0,19 | 0,67 |
| Depressed | 148 | (1,3) |  | 17,6 | 14,2 |  | 0,49 | 0,62 |
| Feeling at peace | 146 | (2,7) |  | 29,5 | 7,5 |  | 0,46 | 0,62 |
| Wasted time | 147 | (2,0) |  | 79,6 | 8,8 |  | 0,28 | 0,66 |
| Personal affairs | 145 | (3,3) |  | 78,6 | 3,4 |  | 0,17 | 0,68 |
|  |  |  |  |  |  |  |  |  |

POS= Palliative care Outcome Scale

Cronbach’s alpha (95% CI) for the 10 POS items on admission was 0.67 (0.59-0.73)
