# Supplementary material for: Validity, reliability and responsiveness to change of the Italian palliative care outcome scale: a multicenter study of advanced cancer patients
Source: BMC Palliat Care. 2016 Feb 26;15:23. doi: 10.1186/s12904-016-0095-6 (PMC4768331; doi:10.1186/s12904-016-0095-6)
Supplement: Additional file 5: — Agreement POS scores self-assessed by patients and assessed by staff at T1 (6 days after admission). (DOCX 19 kb) [file 12904_2016_95_MOESM5_ESM.docx]

Additional file 5: Agreement POS scores self-assessed by patients and assessed by staff at T1 (6 days after admission)

|  |  | Patients | Staff | agreement | | | | |
| --- | --- | --- | --- | --- | --- | --- | --- | --- |
|  | No. | Mean (SD) | Mean (SD) |  | Agreement (%) | agreement within one score (%) | Weighted kappa (95%CI) | Spearman correlation |
|  |  |  |  |  |  |  |  |  |
| Pain | 110 | 1.3 (1.1) | 1.2 (1.1) |  | 57.3 | 93.6 | 0.56 (0.44-0.67) | 0.68 |
| Other symptoms | 110 | 1.3 (1.0) | 1.5 (1.2) |  | 47.3 | 84,5 | 0.44 (0.32-0.56) | 0.58 |
| Anxiety | 110 | 1.7 (1.2) | 1.8 (1.0) |  | 38.2 | 83,6 | 0.32 (0.19-0.45) | 0.43 |
| Family anxiety | 110 | 2.7 (1.1) | 2.5 (1.3) |  | 40.9 | 70,9 | 0.25 (0.11-0.39) | 0.26 |
| Information | 109 | 0.5 (0.9) | 0.7 (1.2) |  | 57.8 | 80,7 | 0.21 (0.05-0.36) | 0.28 |
| Share feelings | 110 | 0.8 (1.0) | 1.1 (1.2) |  | 44.6 | 77,3 | 0.23 (0.10-0.36) | 0.30 |
| Depression | 109 | 1.8 (1.2) | 2.0 (1.2) |  | 43.1 | 76,1 | 0.34 (0.22-0.47) | 0.47 |
| Feeling at peace | 110 | 1.1 (1.0) | 1.6 (1.1) |  | 34.6 | 78,2 | 0.20 (0.09-0.32) | 0.33 |
| Wasted time | 110 | 0.3 (1.0) | 0.2 (0.7) |  | 85.3 | 85,3 | 0.22 (0.01-0.42) | 0.30 |
| Personal affairs | 109 | 0.3 (0.8) | 0.5 (1.1) |  | 76.2 | 76,2 | 0.23 (0.01-0.44) | 0.23 |
|  |  |  |  |  |  |  |  |  |
| POS total score | 109 | 12.0 (5.7) | 13.1 (5.7) |  | - | - | 0,56 (0,41-0,68) * | 0.58 |
|  |  |  |  |  |  |  |  |  |

* one-way Intraclass Correlation Coefficient (ICC)

POS= Palliative care Outcome Scale
